# Within the Wards: Some Probable Causes of Cancer

**Published:** 1889-11-09

**Authors:** 


### Within the Wards.

### SOME PROBABLE CAUSES OF CANCER.

People are distinctly advantaged by being taught to understand the causes of the ills that afflict them. To take such a simple example as a common cold: everybody knows that a "draught" will cause a catarrh of the nose and throat, which, in many cases, will extend to the chest, and in susceptible people will end in an attack of acute bronchitis. The initial cause in this case is exceedingly simple, so simple that many] people despise and treat it with contempt. Yet let everybody consider the worst effect which frequently arises from a draught—namely, acute bronchitis. What does acute bronchitis represent to the sufferer? Several days of pain, fever, sleeplessness, difficult breathing, and extreme weakness] and depression. This is followed by cough, expectoration, continued difficulty of breathing, continued weakness, and great fear of fresh cold. The immediate bad consequences are seldom entirely gone in a month, whilst in many cases they never go away at all, but remain as a " nucleus of tendency, which in many instances steadily increases until chronic bronchitis, with all its weakness, disability, and misery, is permanently established. The beginning of all this mischief was a "draught."

According to Prof. von Esmarch, of Kiel, who knows as much of cancer as any man in the world, and whose authority within the medical profession is second to no man's on this particular subject, many a cancer which has finally destroyed the life of its victim has begun in quite as simple a way as the common cold which is caused by a draught. "It is well known," he says, "that malignant tumours originate in scars"; that is, in healed wounds caused by cuts, burns, ulcers, and the like. The "scar" in certain persons, and under certain circumstances, may take on new growth; the new growth being rapidly produced, poorly nourished, and feebly organised, may break down into a cancerous ulcer, and, exhausting the patient by pain, sleeplessness, weariness, and hopelessness, may finally destroy life. "Old ulcers of the leg and stomach" are specially mentioned by Von Esmarch, as also are old syphilitic ulcers. These may be open for years, or may heal and seem well for years, and yet under unfavourable conditions may develop into life-destroying cancers in the end. "Benign growths may also become transformed into malignant tumours (cancers), for example, warts, naevi" (mother's marks, port wine marks, etc.), and similar growths. This is by no means generally known, though it should be. How may such "benign growths" be prevented from developing into cancers? Principally by the observance of two rules: the general health, in these days the nervous health especially, is to be maintained at a high level; and great care must be taken to avoid irritating the warts or mother's marks, as by friction and the like. So long as they appear quite healthy they must be left severely alone. Few people ever dream how many local and general diseases spring from a permanently depressed nervous system and weak general health. It may be said ordinarily of cancer that "some sources of irritation are usually to be discovered." The irritation may have occurred but once, and that once in susceptible persons may prove to have been quite sufficient. Among the kinds of single "irritation" mentioned by Von Esmarch are "injuries" of all sorts as from blows and falls. Of the permanent irritations are soot, in the case of the chimney-sweeper, tobacco, and, of course, short hot stemmed pipes, paraffin among those who are in the habit of working in paraffin stores, and so on. All these facts point to the observance of the two rules mentioned—viz., to keep the general health good, and to avoid all kinds of things and habits which will irritate the skin, or the mucous membrane, or any other part of the body.

It is an anxious question with many how far cancer may be infective and hereditary; and it certainly is not a question to be answered off-hand. Von Esmarch is both cautious and candid in his utterances on the point. He says, "There is as yet no proof that cancer is an infective disease, and it is by no means probable that it is so." Those, therefore, who are called upon to nurse or to live in the same house with cancer patients need have little anxiety about their own personal safety, at any rate from the point of view of infection. With regard to heredity, or at any rate to predisposition, Von Esmarch speaks less comfortably. "One has always to come back to the assumption," he says, "that a certain predisposition, some diminution in the power of resistance of the tissues, is a necessary factor. Without this, it is impossible to explain how it is that in the great majority of cases in which causes of irritation exist, cancer does not become developed." In other words, how is it that many people can stand all sorts of irritation, blows, black eyes, wounds, skin affections, without ever showing the least sign of cancer, whilst others may develop the disease as the result of a single blow. "One is inclined to look upon the predisposition as inherited without being able to get any further." Here, again, the observant mind is impressed with the importance of the general health. A predisposition to any particular disease is undoubtedly an evil and a disqualification, and is to be so recognised. But it is as easy to make too much of a predisposition as too little. Predispositions may be got rid of; constitutions may be changed. Everybody knows and acts upon the conviction that health may be injured and destroyed. Only a few understand and practically act upon the opposite principle that the constitution may be reformed and built up. It is wonderful what a skilful and painstaking builder may do to restore and preserve even the most rickety and tumble-down of houses. It is equally wonderful what the skilled physician may do for a poor human constitution. The physician, indeed, has usually the advantage of the builder in that, in the case at least of sensible men and women, he can rely upon their own intelligent concurrence and co-operation. With children, especially, it is possible to turn a weak constitution into a strong one, and a diseased into a healthy one. Every medical man of experience and sense has seen numbers of cases of puny and weakly infants transformed by years of careful upbringing into bright, active, and healthy boys and girls. It is probably as possible to get rid of the predisposition to cancer as of any other predisposition—perhaps, indeed, much easier than to get rid of an inherited tendency to consumption. At all events, it is quite certain that sound general health is the best security against local as well as general diseases that a man can possibly have. It is stored wealth and accumulated resources of quite as much, and often of much more, value than landed property or money in consols.

